# A rare case of systemic lupus erythematosus presenting with concurrent myocarditis, central retinal vein occlusion, and deep vein thrombosis

**DOI:** 10.1002/ccr3.9520

**Published:** 2024-10-31

**Authors:** Mehrdad Jafari Fesharaki, Negar Raissi Dehkordi, Zahra Zakeri, Emadoddin Hosseinjani, Nastaran Raissi Dehkordi

**Affiliations:** ^1^ Department of Cardiology, School of Medicine Shahid Beheshti University of Medical Sciences Tehran Iran; ^2^ Cardiovascular Research Center, Shahid Labbafinezhad Hospital Shahid Beheshti University of Medical Sciences Tehran Iran; ^3^ Department of Adult Rheumatology, School of Medicine, Shahid Labbafinezhad Hospital Shahid Beheshti University of Medical Sciences Tehran Iran; ^4^ Department of Cardiology, School of Medicine, Cardiovascular Research Center Shahid Labbafinezhad Hospital

**Keywords:** central retinal vein occlusion, deep vein thrombosis, lupus myocarditis, systemic lupus erythematosus

## Abstract

Although systemic lupus erythematosus may present with broad and variable symptoms, the rare and atypical combined presentation of central retinal vein occlusion, myocarditis, and deep vein thrombosis poses a diagnostic challenge and highlights the need for a comprehensive diagnostic approach. Early diagnosis and prompt treatment help mitigate disease morbidity and mortality, and emphasize the importance of heightened clinical suspicion in complex presentations.

## 1 INTRODUCTION

Systemic lupus erythematosus (SLE) is a chronic autoimmune disease characterized by immune complex deposition and autoantibody production, occurring predominantly in reproductive‐age female patients.^1^ Multiple organ systems may be involved in patients with SLE, including skin, musculoskeletal system, kidneys, heart, and lungs.^2^ Combined central retinal artery and vein occlusion in a patient with systemic lupus erythematosus.^3^, ^4^ While ocular involvement may be a common manifestation of SLE, retinal vein occlusion is an uncommon occurrence in severe vaso‐occlusive retinopathy related to SLE. This condition can appear as occlusion in major vessels, such as the central retinal vessels and the cilioretinal artery, or as extensive microembolization in small vessels. The most severe form of retinopathy in SLE can result in extensive damage to the retina and significant visual impairment^5^ SLE can also lead to myocarditis, an inflammation of the heart muscle caused by immune complex deposition. Myocarditis in SLE can range from asymptomatic to severe heart failure, depending on the extent of inflammation and damage to the heart muscle.^6^ Deep vein thrombosis (DVT) is another known complication of SLE, caused by an increased risk of blood clots associated with the disease.^7^

This case report highlights the unique presentation of SLE in a patient who presented with central retinal vein occlusion, DVT, and myocarditis as the initial symptoms. While each of these manifestations has been previously reported in SLE patients, the occurrence of all three simultaneously is extremely rare. This case highlights the importance of considering the possibility of SLE in patients presenting with seemingly unrelated symptoms, as prompt recognition and treatment can prevent further complications and improve outcomes. It also underscores the need for clinicians to be aware of the wide spectrum of multi‐organ system involvement in SLE and to consider the possibility of the disease in the differential diagnosis of patients presenting with unusual symptoms.

## 2 CASE HISTORY/EXAMINATION

A 35‐year‐old patient presented to the emergency department complaining of blurred vision starting 2 days ago, which had turned into complete visual loss in the right eye the next morning, in addition to swelling in the right leg, and a history of flu‐like symptoms last week. The patient had a history of PTE and DVT following leg trauma 2 years ago and a history of epilepsy with unknown etiology which was under treatment with sodium valproate. According to the family history, the patient's sister had passed away at the age of 20 due to SLE‐related complications. However, it is unclear whether her morbidity was caused by antiphospholipid syndrome (APS), lupus nephritis, or other factors.

Physical exam findings included a malar rash across the nose and cheeks, and unilateral edema of the right lower extremity. Ophthalmologic examination of visual acuity showed no light perception in the right eye, suggestive of occlusion in the central retinal vein. Additional physical exam findings were negative for oral or nasal ulcers, joint pain and swelling, pleural or pericardial rub, and lymphadenopathy.

## 3 METHODS

**FIGURE 1:**
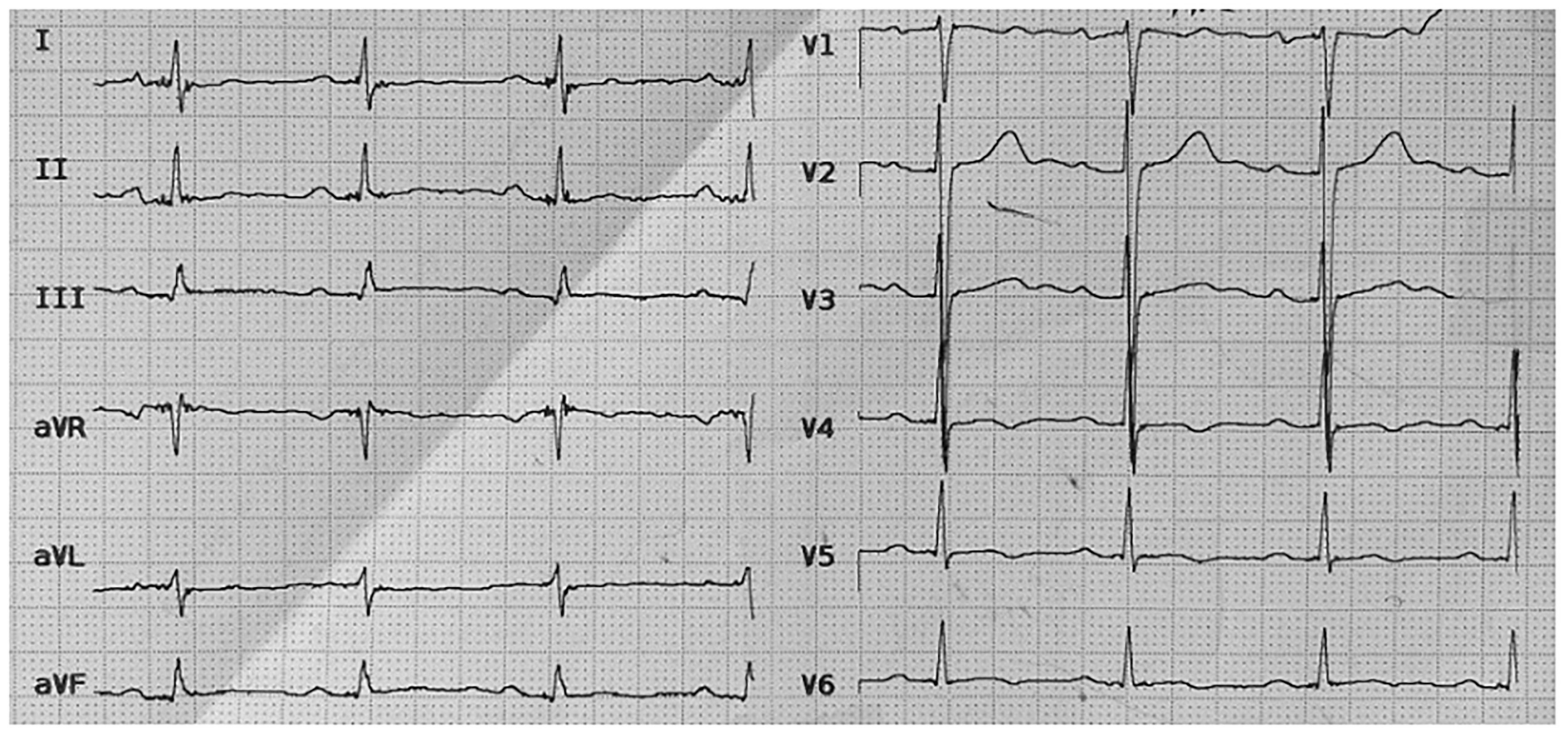
ECG obtained upon admission showed normal sinus rate and rhythm.

Initial blood investigations showed a white blood cell count of 7.8 × 10^3^/μL with 59% neutrophil predominance and 38% lymphocytes, hemoglobin of 6.6 g/dL, and platelet count of 110 × 10^3^/μL. The patient's creatinine levels were within normal range (1.1 mg/dL) and his urine analysis did not show proteinuria. Cardiac troponin level was 8.1 ng/mL and D‐dimer level was significantly increased (8000), while ECG showed normal sinus rate and rhythm with negative T wave in leads V4 to V6 (Figure 1). Doppler sonography of the right lower extremity showed echogenic noncompressible popliteal vein with increased diameter, suggestive of DVT. Follow‐up venous Doppler sonography confirmed the initial diagnosis, and showed decreased diameter in common femoral vein (CFV) and distal part of superficial femoral vein (SFV), suggestive of acute DVT in distal SFV and popliteal vein, and chronic DVT in CFV and proximal SFV. Echocardiography revealed mild‐to‐moderate systolic dysfunction, (EF:40%–45%), severe hypokinesia in the base mid‐inferior wall, posterior wall, and inferoseptal wall, in addition to mild diastolic dysfunction, and moderate mitral regurgitation.

Considering the patient's family history of SLE (in the patient's sister), and DVT in the lower limb, comprehensive rheumatologic lab exams were requested for the patient. Complement levels including C3, C4, and complement CH50 were within normal range. Antinuclear antibody (ANA) was positive in two separate tests, in addition to two positive (>1/160) titers of anti‐double‐strand DNA (anti‐dsDNA). Anti‐cardiolipin Ab (IgG) (ELISA), anti cardiolipin Ab (IgM) (ELISA), B2 glycoprotein Ab (lgG) were negative, while anti‐beta2‐glycoprotein I Ab (IgM and IgG) levels were increased. P.ANCA (anti‐MPO), C.ANCA (anti‐PR3) were negative. A summary of the patient's rheumatologic lab results is shown in Table 1. Coagulation tests for protein C, S, antithrombin III, and factor V Leiden were within normal limits.

**TABLE 1:** A summary of the patient's lab results.

Lab test	Result
White blood cell count	7.8 × 10^3^/μL
Neutrophils	59%
Lymphocytes	38%
Hemoglobin	6.6 g/dL
Platelet count	110 × 10^3^/μL
Creatinine levels	1.1 mg/dL
Urine analysis	No proteinuria
Cardiac troponin level	8.1 ng/mL
D‐dimer level	8000 ng/mL
Complement levels (C3, C4, and CH50)	Within normal range
ANA (antinuclear antibody)	Positive in two separate tests
Anti‐double‐strand DNA (anti‐dsDNA)	Two positive titers (>1/160)
Anti‐cardiolipin Ab (IgG)	Negative
Anti‐cardiolipin Ab (IgM)	Negative
B2 glycoprotein Ab (lgG)	Negative
Anti‐beta2‐glycoprotein I Ab (IgM and IgG)	Increased
P.ANCA (Anti‐MPO)	Negative
C.ANCA (Anti‐PR3)	Negative
Protein C	Within normal limits
Protein S	Within normal limits
Antithrombin III	Within normal limits
Factor V Leiden	Within normal limits

Due to elevated troponin levels, the patient was admitted to the cardiac care unit with a possible diagnosis of acute coronary syndrome and DVT. A coronary angiogram was obtained, which showed normal coronary arteries. Based on the patient's family history of SLE, presenting symptoms, and three positive laboratory findings (ANA, anti‐dsDNA, and anti‐beta2‐glycoprotein I Ab), a diagnosis of SLE was reached. Additionally, the diagnosis of APS was suspected due to an episode of thrombosis and positive anti‐beta2‐glycoprotein I Abs. After ruling out coronary artery disease, a diagnosis of lupus myocarditis was reached, on the basis of clinical findings, laboratory tests (elevated levels of cardiac troponin and anti‐dsDNA), and imaging tests (echocardiogram).

Treatment was initiated with high‐dose corticosteroids, anticoagulant agents, and hydroxychloroquine. Workup for ACS and DVT was initiated, including warfarin 5 mg once daily for anticoagulation, and the patient's INR level was controlled in a range of 3.0–4.0 due to recurring episodes of DVT. An MRI was requested, which did not show any signs of vasculitis and ruled out the need to administer cytotoxic therapies. Regular consultations with cardiology, rheumatology, ophthalmology, and nephrology specialists were conducted to follow the patient's condition.

## 4 CONCLUSION AND RESULTS

The patient's visual acuity remained unchanged, and he was discharged with instructions for continued medical treatment and weekly follow‐up visits with instructions to report any vision deterioration.

## 5 DISCUSSION

The simultaneous occurrence of CRVO, myocarditis, and DVT as initial manifestations of SLE is a rare and previously unreported clinical scenario. A review of the literature reveals that while each of these manifestations can occur in SLE, their concurrent presentation is unique and poses a diagnostic challenge.^8^ Myocarditis, occurring in about 5%–10% of SLE patients,^9^, ^10^ often presents with nonspecific symptoms but can lead to severe complications such as heart failure and arrhythmias.^11^ CRVO, on the other hand, is a rare ocular manifestation of SLE, typically associated with severe vasculitis, leading to significant visual impairment.^5^, ^12^ DVT is a well‐recognized complication in SLE, often linked to APS, but in this case, APS was not definitively diagnosed, which adds another layer of complexity. Given the rarity of this presentation, it is crucial for clinicians to consider SLE in the differential diagnosis when encountering patients with multisystem involvement, even when the symptoms seem unrelated. The importance of interdisciplinary collaboration cannot be overstated, as early diagnosis and prompt treatment can significantly improve patient outcomes and prevent irreversible damage.

## AUTHOR CONTRIBUTIONS

**Mehrdad Jafari Fesharaki:** Conceptualization; investigation; methodology; supervision; writing – original draft; writing – review and editing. **Negar Raissi Dehkordi:** Data curation; investigation; methodology; project administration; resources; writing – original draft; writing – review and editing. **Zahra Zakeri:** Investigation; methodology; validation; writing – review and editing. **Emadoddin Hosseinjani:** Methodology; validation; writing – review and editing. **Nastaran Raissi Dehkordi:** Conceptualization; investigation; methodology; project administration; supervision; writing – original draft; writing – review and editing.

## FUNDING INFORMATION

This work did not receive any funding from private or public sources.

## CONFLICT OF INTEREST STATEMENT

The authors declare that they have no competing interests.

## CONSENT

Written informed consent was obtained from the patient to publish this report in accordance with the journal's patient consent policy.

## Data Availability

Data sharing not applicable‐no new data generated.
